# Antibiotic Stewardship in Pediatric Urinary Tract Infections: Current Evidence and Practical Strategies

**DOI:** 10.3390/antibiotics15070645

**Published:** 2026-06-28

**Authors:** Manar O. Lashkar, Milap C. Nahata

**Affiliations:** 1 Department of Pharmacy Practice, Faculty of Pharmacy, King Abdulaziz University, Jeddah 22254, Saudi Arabia; mlashkar@kau.edu.sa; 2 Institute of Therapeutic Innovations and Outcomes, College of Pharmacy, The Ohio State University, Columbus, OH 43210, USA; 3 College of Medicine, The Ohio State University, 500 West 12th Ave., Columbus, OH 43210, USA

**Keywords:** urinary tract infection, pediatrics, antibiotic stewardship, antimicrobial resistance, pyelonephritis, prophylaxis, intravenous-to-oral, duration of therapy, diagnostic stewardship, uropathogens

## Abstract

**Background/Objectives**: Urinary tract infections (UTIs) are among the most common bacterial infections in children and represent a leading indication for antibiotic prescribing across inpatient, emergency department, and outpatient settings. Despite the availability of multiple international guidelines, prescribing practices for pediatric UTI frequently deviate from evidence-based recommendations in antibiotic selection, route of administration, and duration of therapy. These suboptimal practices contribute to the emergence of resistant uropathogens, including extended-spectrum β-lactamase-producing organisms, and highlight the need for a comprehensive stewardship approach specific to this population. **Methods**: A literature search was performed using PubMed and MEDLINE from January 2000 to May 2026 using the following search terms: urinary tract infection, children, pediatrics, antibiotic stewardship, antimicrobial resistance, diagnosis, treatment, duration, prophylaxis, and intravenous-to-oral transition. Thirteen active international guidelines published between 2011 and 2025 were identified and evaluated with specific emphasis on the integration of antibiotic stewardship principles. Clinical trials, systematic reviews, meta-analyses, and quality improvement studies addressing stewardship-relevant outcomes in pediatric UTI were included. Case reports were excluded. **Results**: Comparative analysis of 13 international UTI treatment guidelines demonstrated substantial variation in diagnostic criteria, treatment duration, and prophylaxis recommendations, with most guidelines predating the SCOUT, STOP, and INDI-UTI randomized controlled trials. Diagnostic stewardship interventions targeting urine collection methods, urinalysis-guided treatment decisions, and avoidance of antibiotic treatment for asymptomatic bacteriuria represented high-impact opportunities to reduce unnecessary antibiotic exposure. Oral antibiotic therapy was as effective as intravenous therapy for most children with pyelonephritis, and early intravenous-to-oral transition was supported by consistent randomized controlled trial evidence. A 5-day oral course may be reasonable for uncomplicated febrile UTI in children demonstrating clinical improvement, supported by the STOP trial, although the SCOUT trial did not meet its noninferiority margin despite a low absolute failure rate; 3 to 5 days was appropriate for uncomplicated cystitis. Antibiotic prophylaxis was not indicated in children with a normal urinary tract following a first febrile UTI and should be reserved for specific high-risk subgroups, with nitrofurantoin as the preferred agent. Formal antibiotic stewardship programs combining prospective audit and feedback, electronic health record integration, and prescriber education demonstrated measurable improvements in prescribing appropriateness for pediatric UTI. Gepotidacin, a first-in-class oral antibiotic approved in 2025 for uncomplicated UTI in female patients aged 12 years and older and weighing at least 40 kg, represented a limited option for eligible adolescents with resistant infections. **Conclusions**: Antibiotic stewardship for pediatric UTI addresses the full clinical pathway from diagnostic stewardship through prophylaxis rationalization. Evidence-based interventions targeting urine collection, urinalysis-guided decision-making, early intravenous-to-oral transition, duration optimization, and selective prophylaxis use can collectively reduce unnecessary antibiotic exposure without compromising patient outcomes. A dedicated stewardship-oriented pediatric UTI guideline, standardized resistance surveillance, and multicenter stewardship program evaluations with patient-centered outcomes are critical research priorities.

## 1. Introduction

Urinary tract infections (UTIs) are among the most common bacterial infections occurring in children. They represent a leading cause of antibiotic prescribing across pediatric inpatient and outpatient settings worldwide [1,2]. Febrile UTI accounts for a large proportion of emergency department visits and hospital admissions in young children [2,3]. If inadequately managed, UTIs may lead to serious complications including urosepsis, renal scarring, and chronic kidney disease. These consequences emphasize the importance of both prompt and appropriate treatment [3,4].

Despite the availability of multiple international treatment guidelines, significant variability in the diagnosis and management of pediatric UTI persists across clinical settings [4,5]. Antimicrobial prescribing patterns for pediatric UTI frequently deviate from evidence-based recommendations. Common suboptimal practices include the selection of broad-spectrum agents when narrower alternatives are appropriate, treatment durations that exceed what evidence supports for uncomplicated infections, prolonged intravenous therapy when oral therapy is feasible, and unnecessary antibiotic prophylaxis use in low-risk populations [6,7,8,9]. These practices have contributed to the emergence of resistant uropathogens, including extended-spectrum β-lactamase (ESBL)-producing organisms, which are increasingly identified as causative agents of community-acquired UTI in children with no established risk factors [10,11].

Antibiotic stewardship programs (ASPs) offer a systematic approach to addressing these prescribing gaps. In pediatric UTI, stewardship interventions have demonstrated measurable improvements in prescribing appropriateness, reductions in antibiotic duration, and successful implementation of intravenous (IV)-to-oral transition protocols [12,13,14]. Nevertheless, to our knowledge, a comprehensive stewardship review specific to pediatric UTI, integrating evidence across the full clinical pathway from diagnostic stewardship through prophylaxis rationalization, has not been previously published in this format.

The past several years have produced a substantial body of new evidence and updated guidance relevant to this field. In 2025, the European Society for Pediatric Infectious Diseases (ESPID) published the first guidelines for complicated UTI in children [15], and the European Association of Urology and European Society for Paediatric Urology (EAU/ESPU) released an updated pediatric UTI guideline [16]. The Infectious Diseases Society of America (IDSA) published its first guideline on complicated UTI, limited to adult patients, further highlighting the absence of equivalent stewardship-oriented guidance for the pediatric population [17]. The national guideline updates from the United Kingdom [18,19], Spain [20], India [21], and regional guidance from Switzerland [22], Saudi Arabia [23], and pan-Asian societies [24] reflect both the evolving evidence base and the challenge of continued heterogeneity in practice that stewardship programs must address [2].

Two major randomized controlled trials addressed treatment duration in pediatric febrile UTI: the SCOUT trial [25] and the INDI-UTI trial [26]. These trials, along with updated systematic reviews and quality improvement initiatives [27,28], have added important new findings. This review examined the current data and practical stewardship strategies for the management of UTIs, with the aim of providing clinicians and antimicrobial stewardship teams with a unified, evidence-based approach to optimize antibiotic use across the full clinical pathway in pediatric patients. This review is directed at clinicians and healthcare professionals involved in the management of pediatric UTI, including pediatricians, infectious disease specialists, pediatric nephrologists and urologists, emergency physicians, clinical pharmacists, and antimicrobial stewardship teams across inpatient, emergency department, and ambulatory care settings.

A literature search was performed using PubMed and MEDLINE from January 2000 to May 2026. Search terms included urinary tract infection, children, pediatrics, antibiotic stewardship, antimicrobial resistance, empiric therapy, diagnosis, intravenous-to-oral transition, duration of therapy, prophylaxis, and diagnostic stewardship, combined using Boolean operators. Articles were included if they addressed antibiotic stewardship-relevant outcomes in pediatric UTI populations and comprised randomized controlled trials, systematic reviews, meta-analyses, clinical practice guidelines, or quality improvement studies. Case reports, conference abstracts, and animal studies were excluded. Reference lists of included articles were screened for additional relevant sources. Thirteen active international guidelines published between 2011 and 2025 were identified and evaluated across stewardship-relevant domains. Evidence was weighed based on study design, with randomized controlled trials, systematic reviews, and meta-analyses prioritized over observational studies, and clinical practice guidelines used to establish current recommended practice.

## 2. Current International Guidelines: Landscape and Stewardship Relevance

A recent narrative review by Alsaywid et al. compared 13 international pediatric UTI guidelines published between 2000 and 2022 and demonstrated variability across diagnostic criteria, urine collection standards, antibiotic selection, duration, and prophylaxis strategies [2]. The current review extends that analysis to include six guidelines published or updated between 2023 and 2025, a period notable for the first dedicated ESPID guidelines on complicated UTI in children [15], an updated EAU/ESPU pediatric UTI guideline [16], and the first IDSA guideline on complicated UTI [17]. These publications are evaluated here with specific emphasis on antibiotic stewardship principles. Table 1 presents a condensed comparison of 13 active international guidelines; the full comparison across all stewardship-relevant domains is provided in Supplementary Table S1. The stewardship focus of each guideline was rated as strong, moderate, or limited. To make these ratings transparent and reproducible, we applied an operational framework developed for this review and informed by established antimicrobial stewardship principles [29]. Ratings were assigned based on the presence and specificity of guidance across six stewardship-relevant domains: (1) diagnostic stewardship and urine collection standards; (2) culture-based de-escalation; (3) duration optimization differentiated by infection type; (4) defined IV-to-oral transition criteria; (5) prophylaxis restriction with explicit indications; and (6) integration of local antibiogram data. A strong rating was assigned when explicit guidance was present in four or more domains, a moderate rating when two to three domains were addressed, and a limited rating when fewer than two domains were present or the guideline focused primarily on pharmacotherapy without stewardship-specific guidance. The domain-level basis for each rating is provided in Supplementary Table S1. Three themes emerge from this analysis.

### 2.1. Diagnostic Variability as a Stewardship Driver

Significant variation exists across guidelines in CFU thresholds, accepted urine collection methods, and the role of urinalysis in guiding empiric treatment decisions. Accepted CFU thresholds range from any growth on suprapubic aspiration to 100,000 CFU/mL or more on midstream clean catch [2,15]. Overly permissive diagnostic criteria contribute to overdiagnosis and unnecessary antibiotic exposure, while excessively stringent thresholds risk undertreating true infections in young infants. Most recent guidelines caution against treating asymptomatic bacteriuria, yet implementation remains inconsistent across clinical settings [15,18,22,32]. The variability in practice across professional groups has been documented, with physicians and surgeons demonstrating substantially different approaches to pediatric UTI investigation and management [33].

### 2.2. Treatment Duration: Consensus Without Incorporation of Recent Evidence

Most active guidelines recommend 7 to 14 days for febrile UTI and 3 to 7 days for lower UTI, but few differentiate duration by age, severity, or clinical response. Most guidelines published prior to 2024 do not incorporate findings from the SCOUT [25], STOP [34], or INDI-UTI [26] trials. The Spanish 2024 guideline [20] and ESPID 2025 [15] are notable exceptions. This lag between trial evidence and guideline revision represents a concrete opportunity for stewardship programs to implement evidence-based duration reductions ahead of formal guideline updates.

### 2.3. Prophylaxis: Persistent Gap Between Guidelines and Practice

Most current guidelines have substantially narrowed indications for continuous antibiotic prophylaxis, reserving it for high-grade VUR, bladder and bowel dysfunction, and neurogenic bladder [15,21,22]. Despite this shift, prophylaxis continues to be prescribed in populations where evidence of benefit is absent or marginal [9,35]. Both the NICE 2024 recurrent UTI guideline [19] and the systematic review by Gkiourtzis et al. [36] identify nitrofurantoin and cranberry products as the options with the strongest evidence for recurrent UTI prevention in children.

## 3. Diagnostic Stewardship in Pediatric UTI

Accurate diagnosis is the foundation of appropriate antibiotic use in pediatric UTI. Overdiagnosis leads to unnecessary antibiotic exposure and resistance selection. Underdiagnosis delays treatment and risks renal parenchymal damage in young infants [1,3]. Diagnostic stewardship includes appropriate urine collection, rational interpretation of urinalysis and culture results, and avoidance of antibiotic treatment in the absence of true infection.

### 3.1. Urine Collection

The method used to collect urine directly affects the reliability of culture results. Contaminated specimens are a recognized driver of unnecessary antibiotic prescribing in children [2,37]. Suprapubic aspiration (SPA) is the gold standard for non-toilet-trained infants; any growth is considered significant [18,30]. Transurethral catheterization is an acceptable alternative with lower contamination rates than bag collection [15,18,22]. Clean catch midstream urine is recommended for toilet-trained children [20,21]. Bag collection carries false-positive rates between 88% and 99% and should be restricted to initial screening only [2]. Despite these recommendations, bag collection remains widely used, contributing to false-positive cultures and unnecessary prescribing [3,7].

### 3.2. Urinalysis and Urine Culture

Urinalysis guides empiric antibiotic decisions while culture results are pending. A positive nitrite result supports a working diagnosis of UTI and justifies empiric therapy [2,18]. A negative result for both leukocyte esterase and nitrite has high sensitivity for excluding UTI [18,30]. Pyuria, defined as more than five white blood cells per high-power field, is a required component of UTI diagnosis in most guidelines and is a critical stewardship checkpoint. Its absence with a positive culture should prompt reassessment before treatment is initiated [30,37]. Culture results must be interpreted alongside clinical findings and urinalysis. The widely accepted threshold is 50,000 CFU/mL or more from catheter or SPA specimens, and 100,000 CFU/mL or more from clean catch specimens [18,20,21,30]. A positive culture alone is insufficient to diagnose UTI and justify antibiotic treatment [32,37].

### 3.3. Asymptomatic Bacteriuria and Culture-Positive, Pyuria-Negative Results

Asymptomatic bacteriuria (ASB) is defined as significant bacteriuria in an individual without signs or symptoms of UTI [32]. Treatment of ASB does not reduce symptomatic UTI risk or prevent renal scarring and is associated with replacement of existing flora by resistant organisms [32,35]. Most current guidelines recommend against treating ASB in children without anatomical abnormalities or planned urological procedures [15,18,21,22]. A positive culture without pyuria in children aged 1 to 24 months was associated with significantly lower rates of clinical improvement with antibiotics [37], supporting a pyuria-first approach and withholding treatment pending clinical reassessment in well-appearing children.

### 3.4. Additional Laboratory Tests

Procalcitonin above 0.5 ng/mL has been reported as a reliable marker of acute pyelonephritis and renal scarring risk [2]. Neither C-reactive protein nor procalcitonin is currently recommended as a routine diagnostic test by most guidelines, with primary utility in risk stratification rather than UTI confirmation [18,21]. Use of inflammatory markers to guide decisions about antibiotic route and duration is a promising stewardship approach but requires further prospective evaluation before routine implementation.

### 3.5. Practical Diagnostic Stewardship Recommendations

Bag collection should be restricted to initial screening and not sent for culture when clinical suspicion is high. Empiric treatment should require both a positive urinalysis and a compatible clinical presentation. Culture results must not automatically trigger antibiotic treatment without urinalysis review. ASB should be documented and managed without antibiotics in the absence of specific indications. Stewardship programs should monitor rates of bag specimen use, culture-to-treatment conversion rates, and antibiotic initiation in the absence of pyuria as measurable metrics [13,14,38].

## 4. Empiric Antibiotic Selection Through an Antibiotic Stewardship Lens

Empiric antibiotic selection is among the key prescribing decisions for pediatric UTI. The choice of agent must balance adequate coverage of the most likely uropathogens while avoiding unnecessary broad-spectrum antibiotic use [1,3]. Several factors influence empiric selection, including the type and severity of infection, the patient’s age, clinical setting, prior antibiotic exposure, urological history, and local antimicrobial resistance patterns [2,39].

### 4.1. Uropathogens and Resistance Patterns

*Escherichia coli* remains the predominant causative organism in pediatric UTI, accounting for approximately 80% to 85% of community-acquired infections [1,2]. Other Gram-negative organisms including *Klebsiella pneumoniae*, *Proteus mirabilis*, and Enterobacter species account for a smaller proportion of cases. Gram-positive organisms including *Enterococcus faecalis* and *Staphylococcus saprophyticus* are less common but clinically important, particularly in neonates and boys [2,30]. Ampicillin resistance among *E. coli* strains exceeds 40% to 50% in most settings, making it unsuitable for empiric use [2,39]. Trimethoprim-sulfamethoxazole resistance exceeds 20% in many regions, which is the threshold above which empiric use is not recommended [2,40]. Nitrofurantoin retains favorable activity against *E. coli* with consistently low resistance rates and is appropriate for empiric treatment of uncomplicated cystitis in children older than one month [2,30]. Local antibiograms specific to the clinical setting must guide empiric selection and cannot be substituted by national or international resistance data [2,6].

### 4.2. Matching Empiric Agent to Infection Type

A stewardship-aligned approach requires matching the antibiotic spectrum to the type and severity of infection. For uncomplicated lower UTI in toilet-trained children, nitrofurantoin or a first-generation cephalosporin is appropriate where local resistance rates are below 20% [2,18,30]. In young children, the clinical distinction between cystitis and pyelonephritis is often difficult, as fever is frequently the primary presenting symptom of UTI regardless of the site of infection. For this reason, nitrofurantoin must not be used empirically in febrile young children, as it does not achieve adequate renal parenchymal concentrations. Trimethoprim-sulfamethoxazole is suitable for definitive therapy once culture results are available, or empirically where local resistance is known to be low [2,22]. Amoxicillin-clavulanate is an alternative for uncomplicated lower UTI in children with a first episode and no recent antibiotic exposure; resistance rates are substantially higher in children with recurrent infections or prior antibiotic use [40]. For febrile UTI and suspected pyelonephritis, oral third-generation cephalosporins including cefixime and cefpodoxime are appropriate first-line agents in children tolerating oral medications without risk factors for resistant organisms [18,20,22]. Intravenous aminoglycosides or third-generation cephalosporins are appropriate for children requiring parenteral therapy [15,30]. Table 2 summarizes empiric agent selection by infection type, age, and clinical setting.

### 4.3. Risk Stratification for Resistant Organisms

Identifying children at risk for ESBL-producing organisms is a critical stewardship function. Risk factors include age under one year, prior antibiotic exposure within three months, recurrent UTI, cephalosporin prophylaxis, recent hospitalization, neurological disease, implanted devices, and clean intermittent catheterization [10,11]. Children with one or more risk factors warrant broader empiric coverage pending culture results. For suspected ESBL cystitis, nitrofurantoin retains activity against most ESBL-producing organisms and is an appropriate empiric option [2,30]. For suspected ESBL pyelonephritis, carbapenem therapy is generally required, with aminoglycosides as an alternative in selected cases [15,30].

### 4.4. Fluoroquinolones—Restricted Use

Fluoroquinolones are not recommended as first-line empiric agents for pediatric UTI. Concerns include musculoskeletal adverse effects and the need to preserve this class for infections where alternatives are limited [41,42]. The AAP updated its position in 2016, acknowledging expanded indications in children and noting that long-term musculoskeletal adverse effects were not demonstrated in pediatric studies, while still recommending limited use [41]. Ciprofloxacin is approved for complicated UTI caused by *E. coli* in children aged one year and older. Its use should be reserved for infections caused by *Pseudomonas aeruginosa* or multidrug-resistant Gram-negative organisms when no oral alternative is available [41,42]. Early work by Alghasham and Nahata documented the long-standing caution around fluoroquinolone use in this population [43].

The agents discussed in the following subsections, including gepotidacin, fosfomycin, and beta-lactam/beta-lactamase inhibitor combinations, are not recommended for routine empiric pediatric UTI therapy. They are presented here as special-case options reserved for selected clinical scenarios involving resistant organisms or specific patient characteristics.

### 4.5. Gepotidacin—A New Option for Adolescents

Gepotidacin, a first-in-class oral triazaacenaphthylene antibiotic that inhibits bacterial DNA replication by a distinct mechanism, received FDA approval in March 2025 for uncomplicated UTI in female adult and pediatric patients aged 12 years and older and weighing at least 40 kg [44]. The EAGLE-2 and EAGLE-3 phase 3 trials enrolled patients aged 12 years and older at 219 centers worldwide and demonstrated non-inferiority or superiority to nitrofurantoin, with activity retained against ESBL-producing *E. coli* and organisms resistant to fluoroquinolones and trimethoprim-sulfamethoxazole [44]. Given these criteria, gepotidacin should be considered as a limited option for eligible adolescent females with resistant uncomplicated cystitis where standard agents are not appropriate, rather than a broad pediatric stewardship recommendation. Data in children younger than 12 years or below the weight threshold are not available.

### 4.6. Fosfomycin

Fosfomycin has an established role in pediatric UTI caused by resistant uropathogens including ESBL-producing and *Enterococcus* species [45]. The oral formulation achieves high urinary concentrations and has been used for resistant uncomplicated cystitis in children where other oral agents are unavailable [45]. The intravenous formulation received FDA approval in November 2025 for complicated UTI in adults, with no known cross-resistance to other antibiotic classes [46]. Pediatric data for the intravenous formulation remain limited.

### 4.7. Beta-Lactam/Beta-Lactamase Inhibitor Combinations for Resistant Infections

Ceftazidime-avibactam and ceftolozane-tazobactam are reserved for confirmed resistant infections including carbapenem-resistant Enterobacteriaceae and multidrug-resistant *Pseudomonas aeruginosa* where standard agents have failed [47]. Evidence in pediatric UTI is limited to small observational studies. Prescribing these agents requires infectious disease consultation and stewardship oversight [47].

### 4.8. De-Escalation—A Core Stewardship Principle

**Figure 1:**
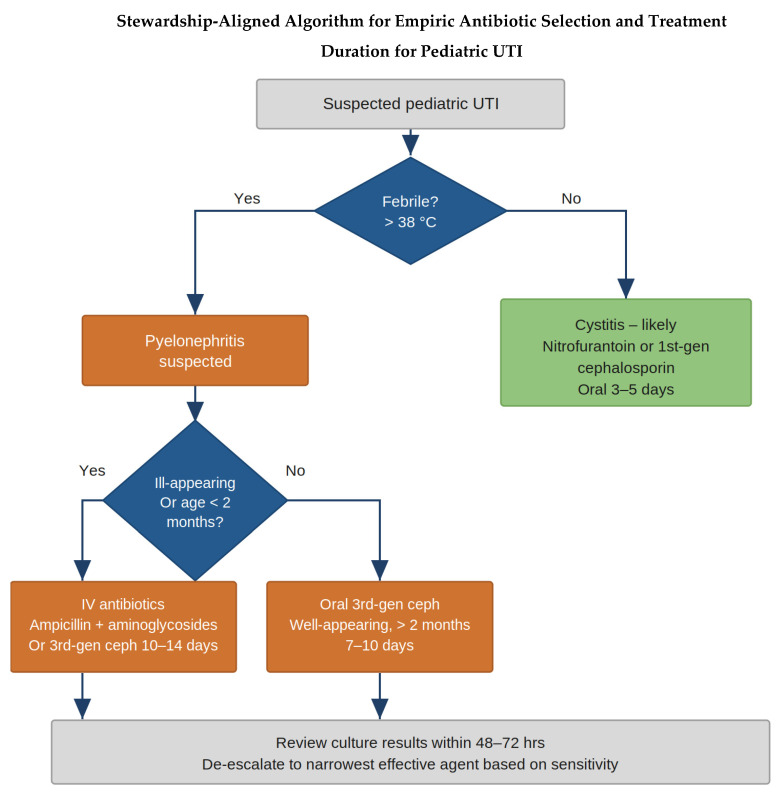
Stewardship-aligned empiric antibiotic selection algorithm for pediatric urinary tract infection. Empiric therapy should be reviewed and adjusted based on urine culture and sensitivity results within 48 to 72 h of initiation. For specific antibiotic dosing, refer to Table 2 and institutional formularies. 3rd–gen ceph, third-generation cephalosporin; IV, intravenous; UTI, urinary tract infection.

Once culture results are available, the empiric regimen should be reviewed and narrowed to the agent with the most appropriate spectrum, most favorable safety profile, and lowest resistance risk [6,12,48]. Studies of outpatient prescribing for pediatric UTI have identified substantial rates of continuation of broad-spectrum agents when narrower alternatives are susceptible [48,49]. Stewardship programs should implement systematic culture review processes that prompt de-escalation within 48 to 72 h of results. Figure 1 presents a stewardship-aligned algorithm for empiric antibiotic selection.

## 5. Intravenous-to-Oral Transition Stewardship

Two related but distinct stewardship strategies are addressed in this section. The first is initial oral therapy, in which oral antibiotics are administered from the outset without any intravenous course. The second is intravenous-to-oral step-down, in which intravenous therapy is started and then converted to oral antibiotics once defined clinical criteria are met. Both approaches reduce intravenous antibiotic exposure and its associated risks, and the evidence supports each in appropriately selected children. Initial oral therapy is preferred for well-appearing children who meet clinical eligibility criteria, whereas IV-to-oral step-down applies to children who require initial parenteral treatment but can transition to oral antibiotics once clinically stable.

### 5.1. Historical Practice and Its Limitations

For decades, pediatric pyelonephritis was managed with prolonged IV antibiotic courses followed by oral step-down only after sustained clinical improvement, extending hospital stay and IV line-associated risks without demonstrated benefit over oral therapy alone [50,51,52,53]. Three randomized controlled trials established the foundation for early oral step-down. Hoberman et al. randomized 306 febrile children aged 1 to 24 months to oral cefixime alone or IV cefotaxime followed by oral cefixime; renal scarring rates at 6 months were equivalent [50]. Montini et al. randomized 502 children aged 1 month to 7 years to oral amoxicillin-clavulanate or IV ampicillin plus gentamicin followed by oral amoxicillin-clavulanate; scarring rates at 12 months were similar [51]. Bocquet et al. found equivalent clinical cure and renal scarring rates between IV ceftriaxone followed by oral cefixime and oral cefixime alone [54]. A Cochrane systematic review of 27 randomized controlled trials enrolling more than 3100 children confirmed that oral antibiotic therapy was as effective as IV-to-oral sequential regimens for preventing renal damage, achieving clinical cure, and preventing recurrence [52]. ASPs now identify early IV-to-oral transition as a high-yield, low-risk intervention in children with febrile UTI [8,12].

### 5.2. Criteria for IV-to-Oral Transition

Broadly accepted criteria for step-down include sustained afebrile status for at least 24 h, tolerance of oral intake, absence of vomiting, and demonstrable clinical improvement [8,15]. Children with concurrent bacteremia, immunocompromised status, obstructive uropathy, or those requiring agents without reliable oral bioavailability may require prolonged IV therapy on an individualized basis. Neonates younger than 28 days are generally excluded from early oral step-down protocols. Table 3 summarizes clinical criteria for and contraindications to early IV-to-oral transition in children with febrile UTI. Third-generation oral cephalosporins and trimethoprim-sulfamethoxazole are appropriate step-down agents when susceptibility is confirmed [53]. Nitrofurantoin must not be used for pyelonephritis step-down as it does not achieve adequate renal tissue concentrations.

### 5.3. Stewardship Program Impact

Brady et al. found that median IV therapy duration varied widely across United States pediatric centers, with a significant proportion of children receiving IV antibiotics beyond clinical eligibility for oral step-down, a finding not explained by differences in disease severity [53]. Newland et al. demonstrated that ASP interventions targeting IV-to-oral conversion were associated with reduced antibiotic days and shorter hospital stays without increasing readmission or treatment failure [55]. ASPs that integrate automatic IV-to-oral switch prompts into the electronic health record, linked to local antibiogram data and pharmacist-driven conversion alerts, demonstrated measurable reductions in IV antibiotic duration without increasing adverse outcomes [8,12,14,55]. Prescriber education on oral bioequivalence data is a documented component of effective and sustained IV-to-oral stewardship programs [12,55].

## 6. Duration of Therapy Optimization

### 6.1. Guideline Recommendations and the Stewardship Case for Shorter Courses

Most active guidelines recommend 7 to 14 days for febrile UTI and 3 to 7 days for lower UTI, with prescribing in practice frequently defaulting to the longer end without evidence of additional benefit [1,15,21,27,30]. Prolonged courses increase adverse drug effects, promote resistant organism selection, and reduce adherence [27,56]. Afolabi et al. found that longer treatment courses were not associated with lower recurrence rates in pediatric UTI, supporting the principle that extended duration does not confer additional benefit in uncomplicated infections [56].

### 6.2. Evidence from Recent Randomized Controlled Trials

The SCOUT trial randomized children aged 2 months to 10 years who demonstrated clinical improvement after 5 days of antimicrobials to a further 5 days of treatment or placebo [25]. Treatment failure occurred in 0.6% versus 4.2% respectively; non-inferiority was not demonstrated, though the absolute failure rate in the short-course group was low and the authors noted short-course therapy as a reasonable option for children with clinical improvement after 5 days [25].

The STOP trial randomized 5-day versus 10-day oral amoxicillin-clavulanate in children aged 3 months to 5 years with uncomplicated febrile UTI [34]. The 5-day course was non-inferior for UTI recurrence within 30 days, providing the strongest evidence to date that 5 days is sufficient for uncomplicated febrile UTI in otherwise healthy young children [34].

The INDI-UTI trial randomized individualized duration (stopped 3 days after clinical improvement with a 4-day minimum) versus standard 10-day treatment in children aged 3 months to 12 years [26]. Median duration in the individualized group was 5.3 days. Non-inferiority was not met at the pre-specified margin, but the individualized group had significantly fewer antibiotic days and fewer antibiotic-related adverse events without difference in serious adverse events [26].

A meta-analysis of nine trials by Mueller et al. found no significant difference in UTI rates at end of therapy between short-course (2–5 days) and standard-course (6–14 days) treatment in children, with shorter courses appearing reasonable for afebrile children and further trials needed for febrile subgroups [27]. Tramper-Stranders noted that the current evidence points toward shorter courses being safe for most children with uncomplicated febrile UTI while acknowledging that optimal duration has not been definitively established for all subgroups [28].

### 6.3. Practical Stewardship Recommendations

A 5-day course may be reasonable for uncomplicated febrile UTI in children who show clinical improvement. This is supported by the STOP trial [34]. The SCOUT trial did not meet its pre-specified noninferiority margin, so clinician judgment remains important [25]. A 3 to 5-day course is appropriate for uncomplicated cystitis in afebrile children [27,30]. Children with complicated UTI, urological abnormalities, or persistent fever beyond 48 to 72 h warrant individualized duration with specialist input [15]. Hawkins et al. identified duration optimization at emergency department discharge as a high-impact stewardship target, with a significant proportion of children receiving courses exceeding guideline recommendations [57]. Stewardship programs should audit duration as a routine metric, with electronic health record alerts flagging prescriptions exceeding recommended durations [13,57].

## 7. Prophylaxis Stewardship

### 7.1. Background

Antibiotic prophylaxis for recurrent UTI prevention has been one of the most debated areas in pediatric urology and nephrology. The evidence supporting broad prophylaxis use has been substantially revised, and most current guidelines now recommend a selective and restrictive approach [15,21,22].

### 7.2. Randomized Controlled Trial Evidence

The RIVUR trial enrolled 607 children aged 2 months to 6 years with grade I to IV VUR randomized to trimethoprim-sulfamethoxazole prophylaxis or placebo for 24 months [58]. Prophylaxis reduced UTI recurrence by approximately 50% but did not reduce renal scarring, and trimethoprim-sulfamethoxazole resistance was significantly higher in the prophylaxis group at breakthrough UTI [58]. The PRIVENT trial enrolled 576 children aged 0 to 18 years randomized to trimethoprim-sulfamethoxazole or placebo for 12 months [59]. Prophylaxis modestly reduced recurrent UTI without preventing renal scarring, with benefit most pronounced in children with grade III to IV VUR and bladder and bowel dysfunction [59]. Both trials raised important stewardship concerns. Prophylaxis reduces recurrence in selected children but does so at the cost of promoting antimicrobial resistance.

### 7.3. Systematic Review and Meta-Analytic Evidence

The Williams and Craig Cochrane review of 16 studies enrolling 2036 children found that antibiotics modestly reduced repeat symptomatic UTI compared with placebo, though the risk of UTI caused by a resistant organism was approximately 2.4 times greater in the prophylaxis group [35]. Gkiourtzis et al. analyzed 23 randomized controlled trials enrolling 3335 children and found that nitrofurantoin and cranberry products were associated with the lowest odds of symptomatic UTI [36]. Nitrofurantoin demonstrated the most favorable evidence for UTI incidence reduction, and trimethoprim-sulfamethoxazole showed higher resistance emergence. No prophylaxis option reduced renal scarring [36].

### 7.4. Prophylaxis in High-Risk Subgroups and Non-Antibiotic Options

Prophylaxis is not indicated in children with a normal urinary tract following a first febrile UTI [21,22,30]. Current guidelines support selective use for children with high-grade VUR (grades III to V), bladder and bowel dysfunction, neurogenic bladder, and recurrent febrile UTI despite adequate treatment [15,21,22]. Chamberlin et al. found that prophylaxis was not associated with significant UTI risk reduction in children with isolated prenatal hydronephrosis, supporting restriction in this population [9]. Non-antibiotic strategies including cranberry products, methenamine hippurate, and bladder and bowel dysfunction management represent important stewardship alternatives. The NICE 2024 recurrent UTI guideline actively encourages these options before antibiotic prophylaxis is initiated [19,22,36].

### 7.5. Stewardship Concerns and Practical Recommendations

Children receiving prophylaxis have higher rates of resistant uropathogens at breakthrough UTI, limiting treatment options when infection occurs [35,36,58]. Trimethoprim-sulfamethoxazole prophylaxis is associated with higher resistance rates compared with nitrofurantoin [36]. Cephalosporin prophylaxis is associated with ESBL emergence and is not recommended [15]. Prophylaxis is appropriate for high-grade VUR, neurogenic bladder, bladder and bowel dysfunction, or recurrent febrile UTI. Nitrofurantoin is the preferred agent. Non-antibiotic strategies are appropriate first considerations. Prophylaxis duration requires review and discontinuation when clinical indications resolve [9,15,19,21,36].

## 8. Antibiotic Stewardship Programs: Implementation and Outcomes in Pediatric UTI

Pediatric UTI is among the most common indications for antibiotic prescribing, making it a high-priority stewardship target [1,3]. Despite evidence-based guidelines, prescribing for pediatric UTI frequently deviates from recommendations in agent selection, route, and duration [6,7,48]. Several studies across clinical settings demonstrate the impact of structured ASP interventions.

Brigadoi et al. evaluated two successive ASP implementations over eight years at a pediatric tertiary center and found measurable improvements in prescribing aligned with guidelines with each intervention, highlighting that sustained ASP engagement over multiple years is required for durable behavior change [12]. Kooner et al. implemented a multicomponent quality improvement initiative targeting antibiotic duration for uncomplicated UTI; the proportion of children prescribed short antibiotic courses aligned with guideline recommendations increased from 13% to over 50% without change in revisit rates [13]. Kieffer et al. similarly found that a significant proportion of children continued empiric antibiotics despite negative cultures, supporting systematic culture review protocols as a high-yield intervention [38]. Same et al. identified discharge prescribing as a persistent gap even at hospitals with established ASPs, recommending targeted discharge stewardship as a complement to inpatient programs [14].

### ASP Strategies and Effectiveness Metrics

Prospective audit and feedback is the most extensively studied ASP strategy in pediatric settings. Newland et al. demonstrated that it was associated with significant reductions in antibiotic use and days of therapy without increasing adverse outcomes [55]. Electronic health record integration, including order sets linked to local antibiogram data, automatic IV-to-oral switch prompts, and culture review alerts, reduced empiric prescribing variation and supported timely de-escalation [8,13,38]. Education integrated with audit and feedback produced more sustained improvements than standalone programs [12,55]. The ROAD Home framework identified discharge-focused ASP strategies as among the highest-yield interventions for reducing antibiotic overuse after hospital discharge [60].

ASPs targeting pediatric UTI benefit from regular audits of prescribing practice. Relevant metrics include rates of guideline-aligned empiric selection, pre-treatment urine culture obtainment, time to IV-to-oral transition, de-escalation rates within 48 to 72 h, antibiotic duration relative to recommendations, and antibiotic discontinuation following negative cultures [12,13,38]. Outcome metrics include readmission rates, treatment failure, and resistant uropathogen rates at follow-up [12,36]. The ambulatory setting, where most pediatric UTIs are managed, remains substantially understudied and represents an important and underdeveloped area for stewardship intervention [48,61].

Stewardship strategies can be organized by clinical setting. In the inpatient setting, prospective audit and feedback, electronic health record IV-to-oral prompts, automatic stop times, culture review alerts, and pharmacist-led discharge review are the most established interventions [12,14,55]. In the emergency department, culture review protocols for negative results, standardized discharge duration defaults, and antibiotic order sets linked to the local antibiogram support appropriate prescribing at the point of care [13,38,57]. In the ambulatory and primary care setting, where most pediatric UTIs are managed and stewardship infrastructure is often limited, practical strategies include delayed prescribing protocols, callback systems for negative culture results, standardized discharge durations for uncomplicated UTIs, and antibiogram-linked outpatient order sets [48,61]. Table 4 summarizes key interventions by setting, with associated outcome measures and implementation considerations.

## 9. Special Populations

Special populations require individualized stewardship approaches that account for age-specific pharmacokinetics, underlying conditions, and resistance risk. The key stewardship principles for each group are summarized below.

### 9.1. Neonates

For the purposes of this review, neonates are defined as infants younger than 28 days of age; young infants aged 1 to 3 months are addressed separately in Section 9.2, as this group represents a distinct risk category with respect to bacteremia risk and management. Neonates carry a substantially higher risk of concurrent bacteremia (10–20% of febrile neonates with UTI) and require mandatory blood culture before antibiotic initiation [15,39]. Oral therapy is not appropriate given limited pharmacokinetic data and the risk of disseminated infection. Parenteral ampicillin plus aminoglycoside or third-generation cephalosporin is the recommended empiric regimen, with de-escalation guided by culture results [15,30]. Non-*E. coli* organisms including *Klebsiella* species and *Enterococcus faecalis* account for a higher proportion of neonatal UTI, and empiric regimens must provide broader coverage than in older children [2,30].

### 9.2. Young Infants

Infants aged 1 to 3 months remain at higher bacteremia risk than older children, though lower than neonates [15,50]. Most guidelines recommend parenteral initial therapy in infants younger than 2 to 3 months, with early oral step-down when clinical improvement is established [22,50]. Oral cefixime has demonstrated equivalent efficacy to parenteral cefotaxime in infants as young as one month [50].

### 9.3. Children with VUR

Children with VUR, particularly high-grade VUR (grades III to V), are at increased risk of recurrent febrile UTI and renal scarring [58,59]. Empiric selection must account for prior culture history and resistance patterns [11,15]. When prophylaxis is prescribed, nitrofurantoin is the preferred agent and duration requires review and discontinuation when clinical indications resolve, as discussed in Section 7.

### 9.4. Children with Bladder and Bowel Dysfunction (BBD)

Children with BBD are at risk of recurrent UTI through incomplete bladder emptying and altered periurethral flora [21,22]. Antibiotic prophylaxis in this group is unlikely to be effective without concurrent management of voiding dysfunction. Behavioral interventions including timed voiding, fluid optimization, and constipation management reduce UTI recurrence and represent non-antibiotic stewardship strategies with documented benefit [21,22].

### 9.5. Children with Neurogenic Bladder

Children with neurogenic bladder are at high risk of recurrent UTI and carry a high prevalence of resistant organisms [15]. Asymptomatic bacteriuria is extremely common in this population and must not be treated in the absence of systemic symptoms or planned urological procedures. This is one of the most actionable diagnostic stewardship targets in this group [15,32]. Empiric selection at the time of symptomatic UTI must be guided by prior culture history [15]. Avoidance of antibiotic treatment for asymptomatic bacteriuria in children with neurogenic bladder is one of the highest-impact diagnostic stewardship interventions in this population. Each unnecessary treatment course poses a potential risk for emergence of increasingly resistant uropathogens and makes future symptomatic episodes more difficult to treat. Stewardship programs caring for these children should establish institutional protocols that define the indications for urine culture and antibiotic treatment and state clearly that bacteriuria without systemic symptoms does not warrant treatment outside the context of planned urological procedures.

### 9.6. Children with Recurrent UTI

Children with recurrent UTI require empiric therapy guided by prior urine culture history rather than standard first-line agents, as resistance patterns differ substantially from first-episode infections [11,40]. Each recurrence warrants a fresh urine culture before treatment initiation.

### 9.7. Immunocompromised Children

Children who are immunocompromised are at increased risk of UTI caused by resistant and opportunistic organisms [15]. Broader empiric coverage is appropriate pending culture results, with prompt de-escalation and infectious disease consultation for resistant infections [15,47].

## 10. Future Directions and Research Gaps

### 10.1. Guideline Development

The most significant gap in the current evidence base is the absence of a comprehensive, pediatric-specific antibiotic stewardship guideline for UTI. The AAP 2011 guideline was retired in May 2021 without a replacement [30]. The IDSA 2025 guideline excludes pediatric patients [17], and neither the ESPID 2025 [15] nor EAU/ESPU 2025 [16] guidelines are stewardship-specific documents. A dedicated, evidence-based, stewardship-oriented guideline incorporating recent trial data and age-specific recommendations across all clinical settings remains a critical unmet need.

### 10.2. Treatment Duration

The SCOUT [25], STOP [34], and INDI-UTI [26] trials used different antibiotics, age ranges, and outcome definitions, limiting direct comparison. None included neonates or children with urological abnormalities. Future trials should prespecify subgroup analyses by age, fever duration, causative organism, and urological anatomy to generate actionable guidance for heterogeneous pediatric populations.

### 10.3. Diagnostic Technologies

Urine culture requires 24 to 48 h, during which empiric therapy is administered without microbiological confirmation. Multiplex PCR panels for uropathogens and resistance genes offer same-day results but have not yet demonstrated consistent impact on antibiotic use reduction or patient outcomes [62]. Urine biomarkers including procalcitonin and interleukin-6 could refine antibiotic route and duration decisions in children at low risk of renal involvement [2,3], but prospective studies integrating biomarker-guided algorithms into ASP protocols are needed before routine implementation.

### 10.4. Resistance Surveillance and Non-Antibiotic Prophylaxis

Current pediatric UTI resistance surveillance is largely institution-specific and geographically fragmented [10,11]. Standardized, prospective, multinational surveillance programs specifically for pediatric uropathogens, integrated into electronic health record clinical decision support tools, are needed. The evidence base for non-antibiotic prophylaxis, including cranberry products, probiotics, and methenamine hippurate, remains limited by small sample sizes and heterogeneous populations [19,35,36]. Large, well-designed randomized controlled trials with standardized UTI definitions and resistance outcomes as primary endpoints are a research priority.

### 10.5. Emerging Agents and Stewardship Research

Gepotidacin and fosfomycin IV represent newly approved options with direct or potential pediatric relevance, as discussed in Section 4. Pivmecillinam and sulopenem etzadroxil/probenecid, both approved for uncomplicated UTI in adult women in 2024, require pediatric pharmacokinetic and safety evaluation before their use can be recommended in children [63,64]. Most published ASP studies for pediatric UTI are single-center quality improvement projects with short follow-up [12,13]. Multicenter, prospective studies with patient-centered outcomes, including treatment failure, readmission, and resistance emergence, are needed across all settings [48,61].

### 10.6. Equity and Global Applicability

The guidelines reviewed in this manuscript were predominantly developed in high-income countries with reliable access to rapid urine culture, local antibiograms, antibiotics, e.g., oral third-generation cephalosporins and nitrofurantoin formulations, and formal stewardship infrastructure. These resources are not uniformly available in low- and middle-income settings, where the burden of pediatric UTI and antimicrobial resistance is substantial [65]. Future stewardship research should address the applicability of these recommendations in resource-limited environments, including simplified diagnostic algorithms for settings without routine culture access, identification of empiric agents appropriate for high-resistance environments, and low-cost stewardship strategies adaptable to primary care with limited pharmacy support [66]. Equity considerations, including disparities in antibiotic access, culture availability, and stewardship resources as well as financial and educational status across socioeconomic and geographic boundaries, should be explicitly incorporated into future guideline development for pediatric UTI.

### 10.7. Artificial Intelligence

A systematic review and meta-analysis of AI and machine learning models for UTI identification reported a pooled AUC of 0.89 in adult populations [67], and a scoping review found UTI and VUR together accounted for 28% of published AI studies in pediatric urology [67]. Application to pediatric stewardship, including prediction of uropathogen identity and resistance patterns before culture results, warrants prospective evaluation.

## 11. Conclusions

Pediatric UTI is one of the most common indications for antibiotic prescribing in children, yet prescribing practices frequently fall short of evidence-based recommendations in antibiotic selection, route of administration, and duration of therapy. This review examined the current evidence and practical stewardship strategies for pediatric UTI management, with the aim of providing clinicians and stewardship teams with a unified approach to optimizing antibiotic use across the full clinical pathway.

Diagnostic stewardship is the essential first step in ensuring appropriate antibiotic use. Urine collection by appropriate methods, rational interpretation of urinalysis results, and avoidance of antibiotic treatment for asymptomatic bacteriuria and culture-positive, pyuria-negative results are high-impact interventions that reduce unnecessary antibiotic exposure without compromising patient outcomes. Empiric antibiotic selection should be matched to infection type, patient age, clinical setting, and resistance risk, with de-escalation based on culture results within 48 to 72 h.

The evidence supporting early intravenous-to-oral transition is substantial and consistent. Oral therapy from the outset, or early transition once clinical improvement is established, is appropriate for most children with pyelonephritis. Duration of antimicrobial therapy is one of the most modifiable stewardship targets. A 5-day course may be appropriate for uncomplicated febrile UTI in children demonstrating clinical improvement, supported by the STOP trial, although the SCOUT trial did not meet its pre-specified noninferiority margin; 3 to 5 days is appropriate for uncomplicated cystitis.

Antibiotic prophylaxis is not indicated routinely following a first febrile UTI in children with a normal urinary tract. When indicated, it should be prescribed with a documented indication, a preferred agent with a favorable resistance profile, and a planned review interval. Non-antibiotic prophylaxis strategies are underutilized and should be considered before antibiotic prophylaxis is initiated. Formal ASP initiatives combining audit and feedback, electronic health record integration, and prescriber education demonstrate measurable improvements in prescribing appropriateness across all clinical settings, with ambulatory care representing a priority for future intervention.

Research priorities include a comprehensive stewardship-oriented pediatric UTI guideline to replace the retired AAP 2011 document, prospective duration trials in specific pediatric subgroups, multinational resistance surveillance, and multicenter ASP evaluations documenting patient-centered outcomes.

## Figures and Tables

**Table 1:** International pediatric UTI treatment guidelines: comparative analysis with antibiotic stewardship focus.

Guideline/Organization	Year	Target Population Age	Recommended Duration	Prophylaxis Recommendation	Stewardship Focus
AAP [30]	2011 (reaffirmed 2016)	2–24 months	7–14 days	Not recommended for grade I–IV VUR after first febrile UTI	Limited
Italian Recommendations [31]	2020	2 months to 3 years	10–14 days	Not routinely recommended after first febrile UTI	Limited
Swiss Consensus [22]	2021	0–16 years	7–10 days upper UTI; 3–5 days lower UTI	Generally not recommended; VUR alone is not an indication	Moderate
SPIDS [23]	2021	3 months to 14 years	7–14 days	Selective; moderate-to-high grade VUR, BBD with VUR, uncircumcised males with VUR	Moderate
Asian Guidelines [24]	2021	Infants and children	7–14 days	Selective; trimethoprim or nitrofurantoin for high-risk patients	Moderate
EAU/ESPU [16]	2021 (updated 2025)	Newborn to adolescent	7–14 days febrile UTI; 3–5 days lower UTI	Non-antibiotic prophylaxis preferred; antibiotic prophylaxis selective for high-risk subgroups	Moderate
NICE [18]	2022 (amended 2025)	Birth to 16 years	7–10 days upper UTI; 3–7 days lower UTI	Not routinely recommended after first UTI; consider in recurrent UTI	Moderate
ISPN [21]	2024	Children 0–18 years	7–10 days pyelonephritis; 3–5 days cystitis	Not indicated with normal urinary tract; recommended for BBD and high-grade VUR	Moderate
NICE Recurrent UTI [19]	2024	Children <16 years and adults without a catheter	Shortest effective course; prophylaxis reviewed every 6 months	Nitrofurantoin or trimethoprim first-line for children; non-antibiotic options including methenamine hippurate actively encouraged	Strong
Spanish Clinical Practice Guideline [20]	2024	Infants and children	Shorter courses supported; acknowledges SCOUT and STOP trial findings	Not routine; prophylaxis not supported in most children	Moderate
ESPID [15]	2025	Children and adolescents <18 years	Individualized by subgroup and clinical response	Recommended for high-grade VUR, spina bifida, BBD, and uncircumcised boys with recurrent UTI	Strong
EAU/ESPU 2025 Update [16]	2025	Newborn to adolescent	Risk-based duration; shorter courses acknowledged for uncomplicated presentations	Non-antibiotic prophylaxis preferred; chemoprophylaxis selective for high-risk subgroups	Moderate
IDSA cUTI [17]	2025	Adults only	5–7 days fluoroquinolone; 7 days non-fluoroquinolone	Not applicable	Strong

Abbreviations: AAP, American Academy of Pediatrics; BBD, bladder and bowel dysfunction; cUTI, complicated urinary tract infection; EAU, European Association of Urology; ESPID, European Society for Pediatric Infectious Diseases; ESPU, European Society for Paediatric Urology; IDSA, Infectious Diseases Society of America; ISPN, Indian Society of Pediatric Nephrology; IV, intravenous; NICE, National Institute for Health and Care Excellence; SPIDS, Saudi Pediatric Infectious Diseases Society; VUR, vesicoureteral reflux. Stewardship focus was rated across six domains (diagnostic stewardship, culture-based de-escalation, duration optimization, IV-to-oral transition criteria, prophylaxis restriction, and local antibiogram integration): strong = four or more domains addressed; moderate = two to three domains; limited = fewer than two domains or primarily pharmacotherapy-focused. The full domain-level comparison is provided in Supplementary Table S1. AAP note: The AAP 2011 clinical practice guideline was retired in May 2021. No replacement guideline has been published to date.

**Table 2:** Suggested empiric antibiotic classes and stewardship considerations for pediatric UTI by infection type, patient age, and clinical setting.

Infection Type	Age Group	Clinical Setting	Recommended Empiric Agent	Route	Duration	Stewardship Direction
Uncomplicated Cystitis	>1 month	Outpatient	Nitrofurantoin	Oral	3–5 days	Avoid in febrile children; not appropriate if pyelonephritis suspected
	>3 months		First-generation cephalosporin (cephalexin)	Oral	3–5 days	Use when nitrofurantoin resistance exceeds 20% locally
	>3 months		Trimethoprim-sulfamethoxazole	Oral	3–5 days	Use for definitive therapy only; avoid empirically if local resistance exceeds 20%
Uncomplicated Pyelonephritis	>3 months, well-appearing	Outpatient	Third-generation cephalosporin (cefixime, cefpodoxime)	Oral	7–10 days	Appropriate if tolerating oral medications and no risk factors for resistant organisms
Febrile UTI	<2 months	Inpatient	Ampicillin plus aminoglycoside or third-generation cephalosporin	IV	10–14 days total; switch to oral when clinically stable	Blood culture recommended; enterococcal coverage with ampicillin essential in this age group
	2 months to 2 years, ill-appearing or unable to tolerate oral	Inpatient	Aminoglycoside or third-generation cephalosporin	IV	Until afebrile and tolerating oral; total 10–14 days	Switch to oral based on culture results; de-escalate within 48–72 h
Complicated UTI	Any age, urological abnormality or recurrent UTI	Inpatient or outpatient	Individualized based on prior culture history and local resistance	IV or oral	10–14 days	Prior culture results essential; avoid standard first-line agents without culture guidance
Suspected ESBL—cystitis	Any age with ESBL risk factors	Outpatient	Nitrofurantoin	Oral	5–7 days	Nitrofurantoin retains activity against most ESBL-producing organisms
Suspected ESBL—pyelonephritis	Any age with ESBL risk factors	Inpatient	Carbapenem (meropenem, ertapenem)	IV	Individualized	Aminoglycoside is an alternative in selected cases; ID consultation recommended
MDR or Pseudomonas infection	Any age, known risk factors	Inpatient	Ceftazidime, piperacillin-tazobactam, or carbapenem	IV	Individualized	Reserve fluoroquinolones for confirmed Pseudomonas with no oral alternative; ID consultation recommended

**Abbreviations:** ESBL, extended-spectrum β-lactamase; ID, infectious diseases; IV, intravenous; MDR, multidrug-resistant; UTI, urinary tract infection. **Notes:** Empiric antibiotic selection should always be guided by local antimicrobial resistance patterns and adjusted based on urine culture and sensitivity results within 48–72 h of initiation. Duration recommendations refer to the total antibiotic course, including any IV-to-oral transition period. For specific antibiotic dosing regimens in pediatric patients, refer to institutional formularies or established pediatric dosing references.

**Table 3:** Clinical criteria for and contraindications to early IV-to-oral transition in children with febrile UTI [8,15].

Criteria Supporting IV-to-Oral Transition	Contraindications to Early Transition
Afebrile for at least 24 h	Persistent fever beyond 48 to 72 h on appropriate therapy
Tolerating oral intake without vomiting	Age younger than 28 days
Demonstrable clinical improvement	Immunocompromised status
Susceptible organism identified on culture	Concurrent bacteremia
Oral step-down agent with reliable bioavailability available	No reliable oral bioavailability for the required agent
Reliable adherence and follow-up anticipated	Obstructive uropathy

IV, intravenous; UTI, urinary tract infection. Contraindications indicate children for whom early transition should be deferred and individualized rather than absolute prohibitions.

**Table 4:** Key antibiotic stewardship interventions for pediatric UTI by setting, with outcome measures and implementation considerations [12,13,14,38,55].

Intervention	Setting	Outcome Measure	Implementation Consideration
Prospective audit and feedback	Inpatient	Antibiotic days, guideline adherence	Requires dedicated pharmacist or infectious disease specialist time
EHR IV-to-oral switch prompts	Inpatient	IV antibiotic days, length of stay	Needs IT infrastructure and order set build
Culture review alerts	Inpatient/ED	De-escalation and discontinuation rates	Requires systematic culture follow-up process
Standardized discharge durations	ED/Discharge	Discharge duration appropriateness	Pharmacist involvement at point of discharge
Antibiogram-linked order sets	Ambulatory	Empiric agent selection, duration	Requires antibiogram linkage and provider buy-in
Delayed prescribing protocols	Ambulatory	Antibiotic initiation rates	Requires clear patient communication
Callback for negative cultures	ED/Ambulatory	Antibiotic discontinuation rates	Requires nurse or pharmacist staffing

ED, emergency department; EHR, electronic health record; IV, intravenous; UTI, urinary tract infection.

## Data Availability

No new data were created or analyzed in this study. Data sharing is not applicable to this article.

## Supplementary Materials

The following supporting information can be downloaded at: https://www.mdpi.com/article/10.3390/antibiotics15070645/s1. Supplementary Table S1: Comparative analysis of 13 international pediatric UTI guidelines across all stewardship-relevant domains.

## Abbreviations

The following abbreviations are used in this manuscript:

AAP	American Academy of Pediatrics
ASB	Asymptomatic bacteriuria
ASP	Antibiotic stewardship program
AUC	Area under the curve
BBD	Bladder and bowel dysfunction
CAP	Continuous antibiotic prophylaxis
CFU	Colony-forming unit
CRP	C-reactive protein
EAU/ESPU	European Association of Urology/European Society for Paediatric Urology
EHR	Electronic health record
ESBL	Extended-spectrum β-lactamase
ESPID	European Society for Pediatric Infectious Diseases
FDA	Food and Drug Administration
IDSA	Infectious Diseases Society of America
ISPN	Indian Society of Pediatric Nephrology
IV	Intravenous
LE	Leukocyte esterase
MDR	Multidrug-resistant
NICE	National Institute for Health and Care Excellence
PCR	Polymerase chain reaction
PRIVENT	Prevention of Recurrent Urinary Tract Infection in Children with Vesicoureteric Reflux and Normal Renal Tracts
RIVUR	Randomized Intervention for Children with Vesicoureteral Reflux
SCOUT	Short Course Outpatient UTI Treatment
SPA	Suprapubic aspiration
STOP	Short Treatment for Outpatient Pyelonephritis
UTI	Urinary tract infection
VUR	Vesicoureteral reflux
