# Supplementary material for: Antibiotic Stewardship in Pediatric Urinary Tract Infections: Current Evidence and Practical Strategies
Source: Antibiotics (Basel). 2026 Jun 28;15(7):645. doi: 10.3390/antibiotics15070645 (PMC13406111; doi:10.3390/antibiotics15070645)
Supplement: Supplementary file 1 [file antibiotics-15-00645-s001.zip › antibiotics-4380896-supplementary.pdf]

Supplementary Materials

Table S1. Comparative analysis of 13 international pediatric UTI guidelines across stewardship-relevant domains.

Guideline / Organization	Country	Year	Target Population Age	UTI Scope	Diagnostic Stewardship	Treatment Route	Recommended Treatment Duration	Prophylaxis Recommendation	Stewardship Focus
AAP [29]	USA	2011 (reaffirmed 2016)	2–24 months	Febrile UTI, first episode	Catheter or SPA; ≥50,000 CFU/mL	Oral or IV based on clinical status	7–14 days	Not recommended for grade I–IV VUR after first febrile UTI	Limited
Italian Recommendations (Italian Society of Pediatric Nephrology and Infectiology) [30]	Italy	2020	2 months to 3 years	First febrile UTI	Catheter or SPA for culture; urinalysis guided empiric treatment	Oral for well-appearing; IV for severely ill or <3 months; early oral switch encouraged	10–14 days	Not routinely recommended after first febrile UTI	Limited
Swiss Consensus (Swiss Pediatric ID / Nephrology / Urology Societies) [22]	Switzerland	2021	0–16 years	All UTI; suspected and recurrent	Catheter or SPA for infants; clean catch for toilet-trained; bag for screening only	Oral preferred; IV for age <60 days or severely ill; early IV-to-oral switch recommended	7–10 days upper UTI; 3–5 days lower UTI	Generally not recommended; VUR alone not an indication	Moderate
SPIDS [23]	Saudi Arabia	2021	3 months to 14 years	Community-acquired UTI; uncomplicated	Catheter for infants and non-toilet-trained; clean catch for toilet-trained	Oral or IV based on age and severity; guided by local resistance patterns	7–14 days	Selective; moderate-to-high grade VUR, BBD with VUR, uncircumcised males with VUR	Moderate
Asian Guidelines (Japanese / Korean / Taiwanese Societies) [24]	Asia	2021	Infants and children	All UTI	SPA or catheter for non-toilet-trained; clean catch for toilet-trained	Oral for uncomplicated; IV for febrile UTI in young infants; early oral switch when appropriate	7–14 days	Selective; trimethoprim or nitrofurantoin for high-risk patients	Moderate
EAU/ESPU [16]	Europe	2021 (updated 2025)	Newborn to adolescent	All pediatric UTI	SPA or catheter for non-toilet-trained; clean catch for toilet-trained; two-step procedure recommended	Oral or IV based on age and severity; early switch encouraged	7–14 days febrile UTI; 3–5 days lower UTI	Non-antibiotic prophylaxis preferred; antibiotic prophylaxis selective for high-risk subgroups	Moderate
NICE [18]	UK	2022 (amended 2025)	Birth to 16 years	All UTI; first and recurrent	Clean catch preferred; catheter or SPA when unavailable; negative LE and nitrite effectively excluded UTI	Oral for 3 months and older; IV for age <3 months or severely unwell	7–10 days upper UTI; 3–7 days lower UTI	Not routinely recommended after first UTI; consider in recurrent UTI	Moderate
ISPN [21]	India	2024	Children 0–18 years	UTI and primary VUR	Catheter or SPA for infants; clean catch for toilet-trained; ≥10,000 CFU/mL significant with strong clinical suspicion	Oral equivalent to IV for non-toxic infants; pyelonephritis treatable orally for 7–10 days	7–10 days pyelonephritis; 3–5 days cystitis	Not indicated with normal urinary tract; recommended for BBD and high-grade VUR	Moderate
NICE Recurrent UTI [19]	UK	2024	Children <16 years and adults without a catheter; age-specific antibiotic dosing provided	Recurrent UTI prevention only	Culture to confirm recurrence before prophylaxis; asymptomatic bacteriuria not to be treated	Antibiotic choice guided by culture results and local resistance patterns	Shortest effective course; prophylaxis reviewed every 6 months	Nitrofurantoin or trimethoprim first-line for children; non-antibiotic options including methenamine hippurate actively encouraged	Strong
Spanish Clinical Practice Guideline (Spanish Pediatric Societies) [20]	Spain	2024	Infants and children	All pediatric UTI	Midstream clean catch for continent children; catheter or SPA for incontinent requiring immediate diagnosis	Oral preferred; IV for severely ill or unable to tolerate oral; early transition emphasized	Shorter courses supported; acknowledged SCOUT and STOP trial findings	Not routine; prophylaxis not supported in most children	Moderate
ESPID [15]	Europe	2025	Children and adolescents <18 years	Complicated UTI only	Culture mandatory before treatment; blood culture for sepsis presentation; subgroup-guided investigation	IV for complicated subgroups; early oral switch when clinically stable	Individualized by subgroup and clinical response	Recommended for high-grade VUR, spina bifida, BBD, and uncircumcised boys with recurrent UTI	Strong
EAU/ESPU 2025 Update [16]	Europe	2025	Newborn to adolescent	All pediatric UTI	Risk-stratified investigation; local sensitivity patterns mandatory for agent selection	Prompt antimicrobial therapy; route based on age and clinical condition	Risk-based duration; shorter courses acknowledged for	Non-antibiotic prophylaxis preferred; chemoprophylaxis	Moderate

Guideline / Organization	Country	Year	Target Population Age	UTI Scope	Diagnostic Stewardship	Treatment Route	Recommended Treatment Duration	Prophylaxis Recommendation	Stewardship Focus
							uncomplicated presentations	selective for high-risk subgroups	
IDSA cUTI [17]	USA	2025	Adults only	Complicated UTI	Not applicable	IV-to-oral switch when clinically improving; oral bioequivalent agents supported	5–7 days fluoroquinolone; 7 days non-fluoroquinolone	Not applicable	Strong

Abbreviations: AAP, American Academy of Pediatrics; BBD, bladder and bowel dysfunction; CFU, colony-forming units; cUTI, complicated urinary tract infection; EAU, European Association of Urology; ESPID, European Society for Pediatric Infectious Diseases; ESPU, European Society for Paediatric Urology; IDSA, Infectious Diseases Society of America; ISPN, Indian Society of Pediatric Nephrology; IV, intravenous; LE, leukocyte esterase; NICE, National Institute for Health and Care Excellence; SPA, suprapubic aspiration; SPIDS, Saudi Pediatric Infectious Diseases Society; UTI, urinary tract infection; VUR, vesicoureteral reflux.

Stewardship focus rating. Ratings were assigned based on the presence and specificity of guidance across six stewardship-relevant domains: diagnostic stewardship and urine collection standards; culture-based de-escalation; duration optimization differentiated by infection type; defined IV-to-oral transition criteria; prophylaxis restriction with explicit indications; and integration of local antibiogram data. Strong = explicit guidance in four or more domains; Moderate = two to three domains addressed; Limited = fewer than two domains present or primarily pharmacotherapy-focused without stewardship-specific guidance.

Note. The AAP 2011 clinical practice guideline was retired in May 2021; no replacement guideline has been published to date.
